# Spontaneous Bilateral Vertebral Artery Dissection as a Rare Cause of Posterior Circulation Stroke in a Young Patient

**DOI:** 10.7759/cureus.65738

**Published:** 2024-07-30

**Authors:** Nikhil Pantbalekundri, Shilpa A Gaidhane, Suprit Malali, Manikanta Nelakuditi

**Affiliations:** 1 Department of General Medicine, Jawaharlal Nehru Medical College, Datta Meghe Institute of Health Education and Research, Wardha, IND

**Keywords:** stroke in the young, posterior circulation stroke, traumatic vertebral artery dissection, spontaneous vertebral artery dissection, vertebral artery dissection

## Abstract

In young patients, ischemic stroke is an uncommon result of vertebral artery dissection (VAD). Damage to the vertebrae can occur suddenly or as a result of trauma. There are no generally recognized recommendations for diagnosis and treatment, and the majority of vague symptoms and delayed presentation provide a significant diagnostic problem. While medical management with anticoagulant or antiplatelet therapy is advised, no successful dual therapy has been documented. Although traumatic dissection is a more frequent cause of posterior cerebral circulation stroke in people under 45 years of age than spontaneous dissection, spontaneous VAD is well-reported and usually treated with anticoagulation. VAD can result in fatal complications such as basilar area infarction, even though it is often asymptomatic. Here is a case of a 37-year-old male who presented with a posterior circulation stroke after bilateral VAD with no evidence of trauma to the neck and no detectable cause suggesting spontaneous etiology.

## Introduction

The basilar artery is an important blood vessel that plays a significant part in the brain's posterior circulation. It forms at the intersection of the medulla oblongata and the pons, where the dual vertebral arteries confluence, providing blood straight to the brainstem and cerebellum. Via its cortical branches, it divides into the paired posterior cerebral arteries and then supplies blood to the thalami, medial temporal, and occipital lobes [1]. Arterial dissection occurs when blood enters along the intima-medial plane through a rupture caused either spontaneously or by trauma. Cervical artery dissections frequently cause acute ischemic stroke in children and young adults. Either spontaneously or as a result of craniocervical trauma, Valsalva maneuvers, vigorous coughing or sneezing, or genetic diseases such as Marfan syndrome, Ehler-Danlos syndrome (EDS) type IV, Turner syndrome, osteogenesis imperfecta (OI) type I, and fibromuscular dysplasia, arterial dissections can occur. The extracranial internal carotid and vertebral arteries are majorly involved in dissection [2]. Computed tomography angiography or magnetic resonance imaging with the fat-saturation sequence of the cervical region exhibits exceptionally high sensitivity and specificity for diagnosing arterial dissections in the cervical region. Treatment options for acute ischemic stroke with vertebral or carotid artery dissection include antiplatelet and anticoagulation therapy which are still up for debate [3]. Although it is not a prevalent cause of stroke in the general population, vertebral artery dissection (VAD) is one of the more common causes of stroke in young individuals [4]. This case study describes a young male who presented with a posterior circulation stroke with no detectable causes, suggesting a spontaneous etiology.

## Case presentation

**Figure 1:**
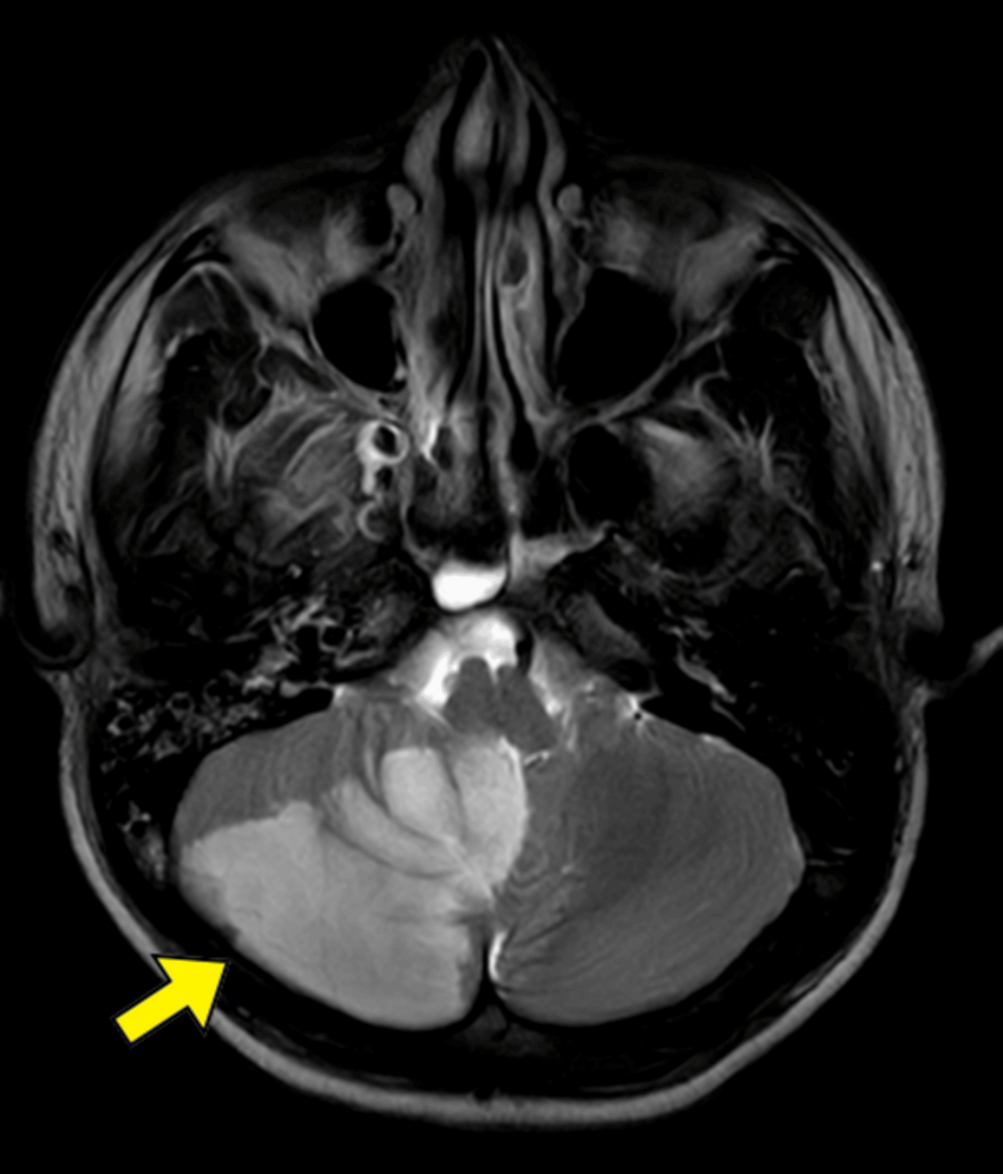
Magnetic resonance imaging (MRI) of the brain showing extensive area of hyperintensity in the right cerebellar hemisphere and mid-brain (yellow arrow).

**Figure 2:**
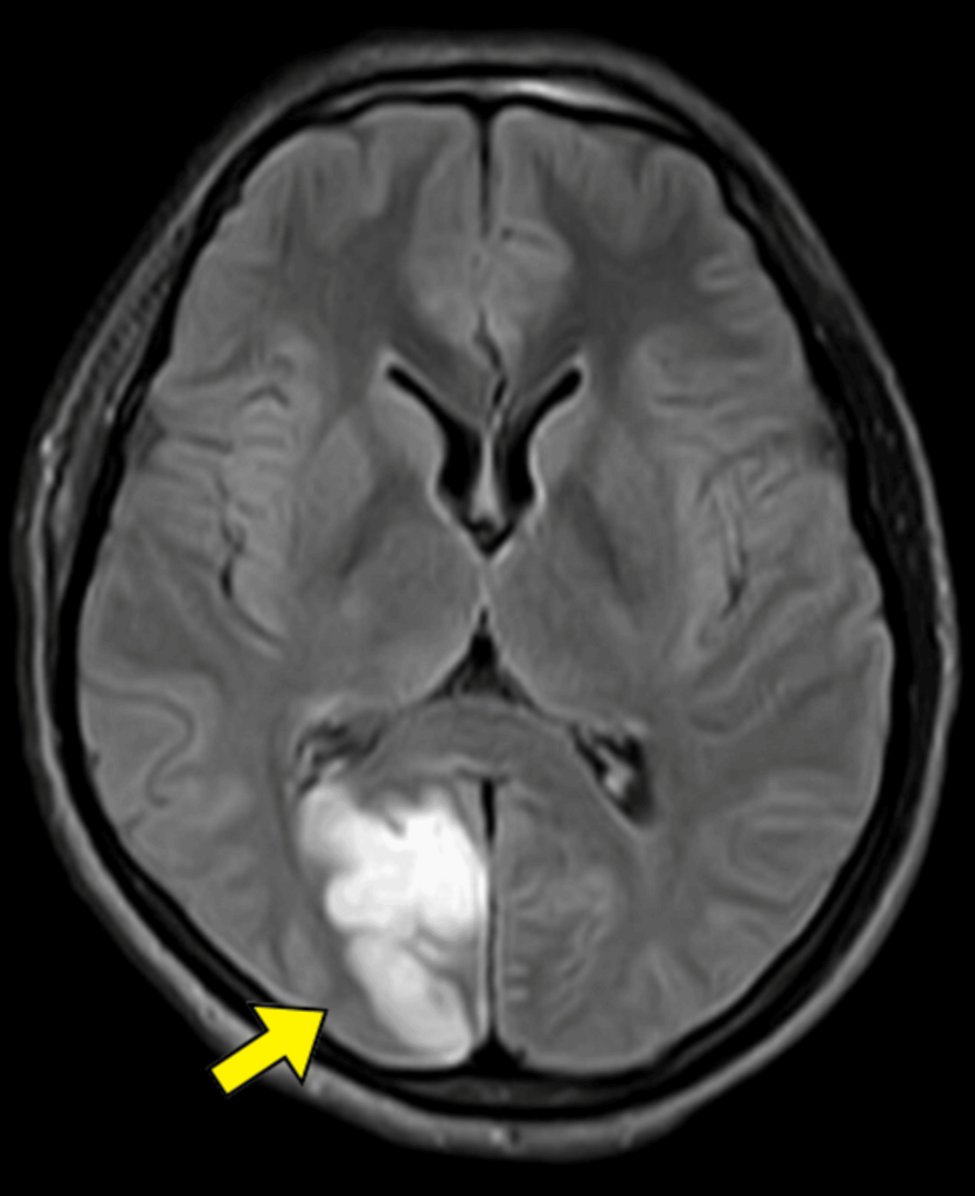
Magnetic resonance imaging (MRI) of the brain showing hyperintensity right occipital lobe (yellow arrow).

A young male was brought into the emergency department with a two-day history of left-sided weakness with numbness of the left side of his body, dizziness, and dysphasia. Over two days, the patient developed difficulty swallowing solids and liquids and weakness on the left side of his body. It was followed by a decline in higher functions per the Glasgow Coma Scale (GCS). The patient had a history of similar complaints of dizziness and transient loss of consciousness one week ago. However, he had no history of recent or remote trauma, diabetes mellitus, dyslipidemia, hypertension, heart disease, connective tissue disorders, immune-compromised states, autoimmune disorders, or drug abuse. He was drowsy at presentation, with a heart rate of 106 per minute and a blood pressure of 90/60 mm of mercury. A poor GCS of E2V2M3 and a National Institute of Health Stroke Scale (NIHSS) score of twelve was noted on neurological examination. Motor examination revealed hypertonia, exaggerated reflexes, and an extensor plantar reflex in the left upper and lower limbs. Blood investigations were done. There was no evidence of hemoglobinopathies on hemoglobin electrophoresis. Following protocol, a computed tomography (CT) scan of the brain was performed; the results showed no signs of bleeding. An axial diffusion-weighted magnetic resonance imaging (MRI) scan revealed large regions of hyperintensity in the right-sided cerebellum and midbrain (Figure 1), and right occipital lobe (Figure 2).

**Figure 3:**
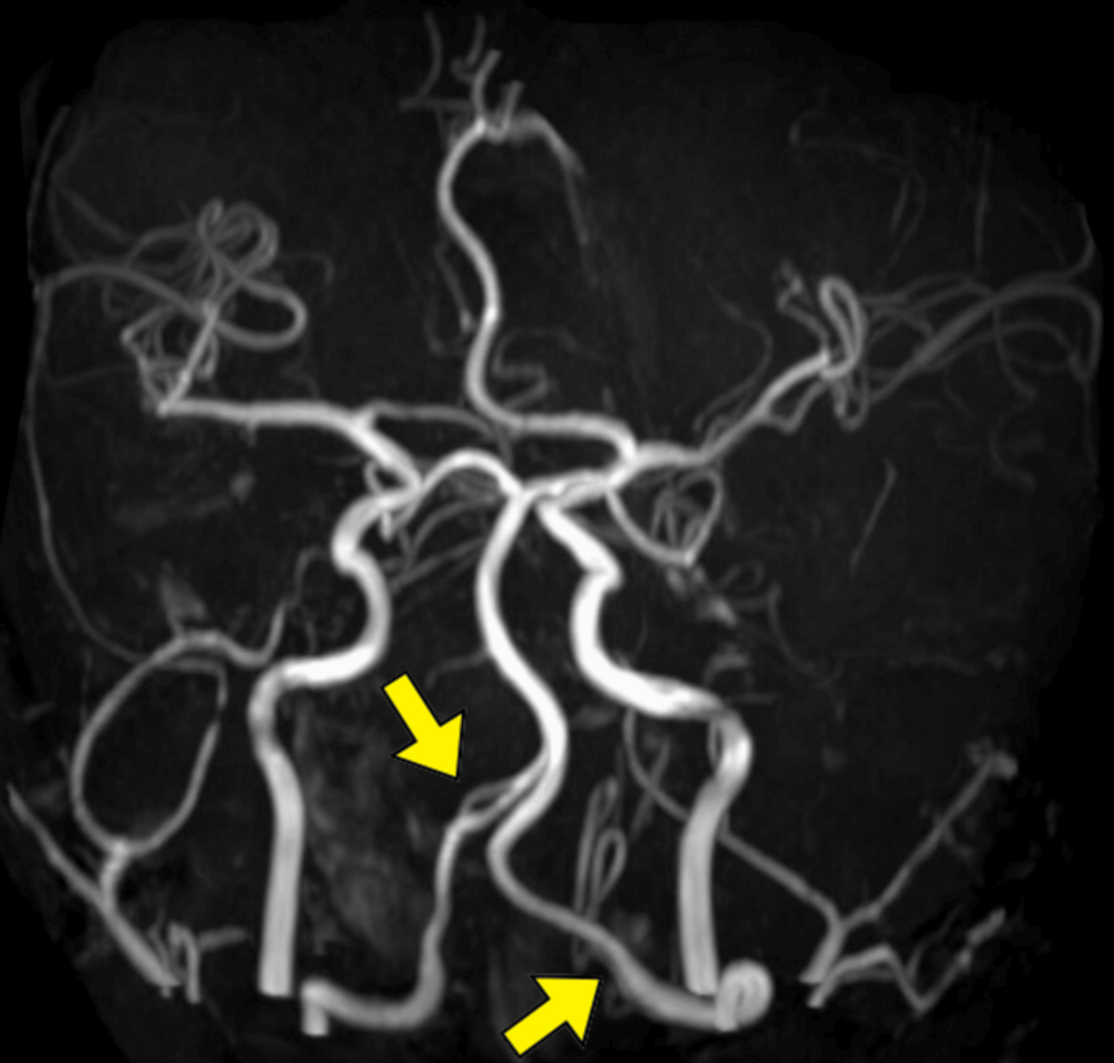
Digital subtraction angiography (DSA) showing bilateral vertebral artery dissection.

A magnetic resonance angiography was done, which revealed a crescent sign in bilateral vertebral arteries, indicating dissection and an average diameter of both internal carotid arteries. After consulting interventional radiology, these findings were confirmed with a digital subtraction angiography (Figure 3). In addition, a flow-dependent flap was seen on the color duplex ultrasonogram of vertebral arteries bilaterally. The patient was continued on supportive management but succumbed on the sixth day of hospitalization.

## Discussion

Dissections of the vertebrae can occur suddenly or as a result of trauma. They are frequently linked to injuries to the head, neck, and cervical spine if they are severe. Significant infarction may result if they are not identified or treated. In contrast to the conventional scenario when the dissection occurs immediately following the traumatic damage, our case presentation shows that it happened exceptionally later [5]. The vertebral arteries ascend to the base of the skull from the subclavian artery on either side and join to form the single basilar artery. The vascular anatomy is relatively uniform. The left vertebral artery is dominant in 70% of people, and up to 10% of people may suffer unilateral hypoplasia, according to research. A dissection most frequently occurs in the distal third of the course of the vertebral artery [6]. A 13% incidence of numerous spontaneous intracranial and extracranial artery dissections was observed in one research [7]. However, another investigation using extensive four-vessel cerebral angiography found that the incidence of these dissections was 22% [8]. It is impossible to draw any relevant comparisons between these articles and the current instance because none addressed the time between the multiple arterial dissections. VAD is more common in patients with hypertension, oral contraceptive use, smoking, connective tissue diseases, migraines, and traumatic events like chiropractic manipulation, falls, violent coughing, sports, or nose-blowing [9].

Numerous genetic diseases can be the cause of spontaneous multiple arterial dissection. The most well-known of these are the monogenic connective tissue illnesses, which include Marfan syndrome, OI type I, and EDS type IV. Other monogenic disorders, such as autosomal dominant polycystic kidney disease, alpha-1-antitrypsin deficiency, and hereditary hemochromatosis, as well as uncommon chromosomal disorders, such as Turner syndrome and William syndrome, have also been linked to sporadic case reports [10]. The autosomal dominant condition known as EDS type IV is brought on by mutations in the COL3A1 gene, which codes for type III collagen. Reduced collagen levels in the blood can cause vascular fragility, which can lead to severe and sometimes deadly outcomes such as aneurysms, arteriovenous fistulae, dissections, and intestinal or vascular ruptures [11]. Another autosomal dominant illness, OI type I, is caused by mutations in the type I collagen-encoding COL1A1 and COL1A2 genes. Some instances linked to heritable connective tissue disorders in earlier reports experienced multiple intracranial and extracranial artery dissections. A critical component of this mechanism might have been vascular fragility in the connective tissue [12]. The most typical clinical manifestations included vertigo, nausea, and dizziness; occipital headache; and unilateral or bilateral neck pain. Along with bilateral internal carotid artery dissection and other multi-vessel compromise symptoms, two patients also had gaze deviation or monocular vision loss. Three cases of locked-in syndrome, one case each of Horner syndrome and Brown-Sequard syndrome, were the least frequent presentations [13]. Because VAD presents in various ways and there are no universal screening protocols that regulatory bodies or academic institutions recognize, it can be challenging to identify and diagnose clinically.

With a sensitivity of 60% for detecting alterations in the brain after artery dissections, cranial magnetic resonance imaging/ magnetic resonance angiography (MRI/MRA) is a noninvasive diagnostic technique [14]. VAD treatment options are pretty limited, and there is an alarming lack of controlled studies in this area. Antiplatelet therapy is being used instead of standard anticoagulation in the treatment of ischemic strokes caused by cervical artery dissections (CAD), according to recent data. While both the CADISS (Cervical Artery Dissection in Stroke Study) and TREAT-CAD (Aspirin Versus Anticoagulation in Cervical Artery Dissection) studies noted a low rate of stroke recurrence, they did not find a statistically significant difference in the efficacy of antiplatelets and anticoagulants. Overall, the choice of medication may depend on the unique risk factors and clinical presentation of each patient, however the STOP-CAD (Antithrombotic Treatment for Stroke Prevention in Cervical Artery Dissection) trial also revealed a nonsignificant tendency favoring anticoagulation in patients with occlusive dissections [15]. When a pseudoaneurysm persists, or in individuals who continue to experience symptoms from thromboembolic episodes or who continue to experience symptoms after starting anticoagulant therapy, intervention or surgery should be considered. Treatment for aneurysms, pseudoaneurysms, and dissection utilizing minimally invasive techniques, including embolization and stent deployment, is also growing in popularity [16]. However, the risk of vascular rupture, dissection, fistula formation, postoperative hemorrhage, and poor wound healing might be increased by intrusive procedures or negligent manipulations, which may raise the mortality rate and necessitate repeat surgery. Although significant neurologic and cognitive impairments, severe strokes, and mortality occur in 4% of people with VAD, the prognosis is often good [17]. Therefore, surgeons must take all reasonable precautions to reduce the possibility of a hazardous event. These precautions include but are not limited to being aware of tissue susceptibility and using extreme caution when performing interventions. Because of the potential for endovascular issues linked to frail veins caused by genetic abnormalities, doctors should carefully weigh the benefits and risks of the intervention. 

## Conclusions

Considering vertebral artery dissection in young patients with stroke is crucial, and early routine imaging should be prioritized before neurological deficits manifest. While spontaneous bilateral internal carotid artery and vertebral artery dissections are rare, they can be a significant cause of stroke in the young population. A thorough diagnostic workup is necessary to exclude rheumatologic causes or connective tissue disorders. Although medical management is a fundamental initial treatment, endovascular repair can be a safe and effective alternative in cases where it proves ineffective, particularly in selected cases.
